# PhTX-II a Basic Myotoxic Phospholipase A_2_ from *Porthidium hyoprora* Snake Venom, Pharmacological Characterization and Amino Acid Sequence by Mass Spectrometry

**DOI:** 10.3390/toxins6113077

**Published:** 2014-10-31

**Authors:** Salomón Huancahuire-Vega, Luis Alberto Ponce-Soto, Sergio Marangoni

**Affiliations:** 1 Department of Biochemistry, Institute of Biology, State University of Campinas (UNICAMP), P.O. Box 6109, 13083-970 Campinas, SP, Brazil; E-Mails: poncesoto@yahoo.com.ar (L.A.P.-S.); marango@unicamp.br (S.M.); 2 Department of Biochemistry and Pharmacology, Faculty of Health Sciences, Medicine School, Union Peruvian University (UPeU), Lima 15, Peru

**Keywords:** PhTX-II, Asp-49 PLA_2_, *Porthidium hyoprora*, myotoxin, edema-forming activity

## Abstract

A monomeric basic PLA_2_ (PhTX-II) of 14149.08 Da molecular weight was purified to homogeneity from *Porthidium hyoprora* venom. Amino acid sequence by *in tandem* mass spectrometry revealed that PhTX-II belongs to Asp49 PLA_2_ enzyme class and displays conserved domains as the catalytic network, Ca^2+^-binding loop and the hydrophobic channel of access to the catalytic site, reflected in the high catalytic activity displayed by the enzyme. Moreover, PhTX-II PLA_2_ showed an allosteric behavior and its enzymatic activity was dependent on Ca^2+^. Examination of PhTX-II PLA_2_ by CD spectroscopy indicated a high content of alpha-helical structures, similar to the known structure of secreted phospholipase IIA group suggesting a similar folding. PhTX-II PLA_2_ causes neuromuscular blockade in avian neuromuscular preparations with a significant direct action on skeletal muscle function, as well as, induced local edema and myotoxicity, in mice. The treatment of PhTX-II by BPB resulted in complete loss of their catalytic activity that was accompanied by loss of their edematogenic effect. On the other hand, enzymatic activity of PhTX-II contributes to this neuromuscular blockade and local myotoxicity is dependent not only on enzymatic activity. These results show that PhTX-II is a myotoxic Asp49 PLA_2_ that contributes with toxic actions caused by *P. hyoprora* venom.

## 1. Introduction

Snakes of the genus *Bothrops* (including *Porthidium* and *Botriopsis*) represent the ophidian fauna of great scientific and medical interest in Brazil, since they are responsible for most cases of deadly snake bites which occur in the country [1,2]. *Porthidium hyoprora* is a representative of this group of venomous snakes; their habitat is the Amazon tropical forest in Brazil, Ecuador, Peru and Colombia [3]. Proteomic analysis of *P. nasutum and P. ophryomegas* snake venom and the characterization of toxins from *P. lansbergii hutmanni* [4,5] have already been performed. However, minimum attention has been paid to the characterization of *P. hyoprora* venom. This snake is named Amazonian hog nose pit viper, whose venom has high phospholipase A_2_ activity and induces drastic local myotoxicity [6].

The phospholipase A_2_ (PLA_2_) superfamily consists of a broad range of enzymes defined by their ability to catalyze the hydrolysis of the middle (sn-2) ester bond of substrate phospholipids [7]. This family of proteins can be found in the mammalian pancreas and also in the venoms of snakes, scorpions and bees. Snake venoms contain plural PLA_2_ isozymes which show wide variety of physiological activities such as neurotoxicity, myotoxicity, cardiotoxicity, platelet aggregation induction or inhibition, edema, hemolysis, anti-coagulation and hypotension [8]. A PLA_2_ may have more than one specific physiological activity, and therefore, it may play multiple roles in the overall effects of envenoming [9].

Significant advances were performed in the efforts to characterize and understand venom PLA_2_; however, many aspects of the structure and function of PLA_2_ present in snake venoms are still a mystery, and new enzymes present in less studied venoms need to be discovered and characterized [10]. A goal of our studies is to characterize the toxins from *P. hyoprora* snake venom, steered by the development and refinement of chromatographic and mass spectrometry techniques, given that the isolation and characterization of individual venom components constitutes the mainstay of toxinology, as a key strategy to dissect and to analyze the complex series of events involved in envenoming [9].

In a previous work, we showed that *P. hyoprora* snake venom is a rich source of PLA_2_ enzymes, and also, PhTX-I PLA_2_ was purified and characterized [6,11]. In the present study, we expand on this pit viper by analyzing one novel myotoxic Asp49 PLA_2_ (PhTX-II) isolated and sequenced by tandem mass spectrometry. Further, our study provides an insight into the biochemical and pharmacological properties of this basic PLA_2_, and suggests that it exerts its myotoxic action by both enzymatic and non-enzymatic mechanisms.

## 2. Results

### 2.1. Purification and Biochemical Characterization of PhTX-II

**Figure 1:**
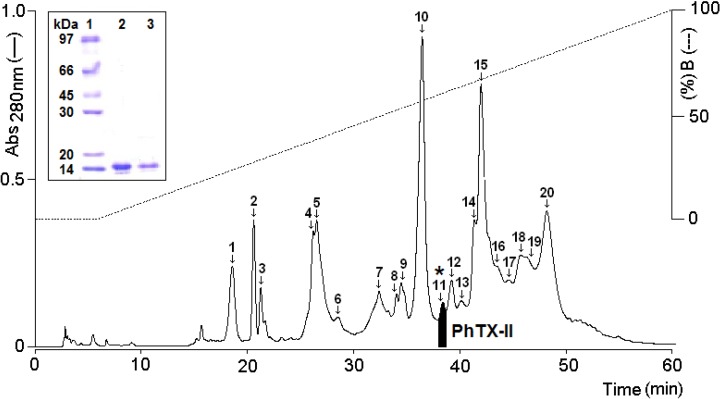
Chromatographic and electrophoretic profile of *Porthidium hyoprora* venom fractioning on a µ-Bondapack C18 column, monitoring elution profile at 280 nm. Emphasized in black is fraction 11 (*) characterized as PhTX-II PLA_2_; Insert: Electrophoretic profile in Tricine SDS-PAGE (1) Molecular mass markers; (2) PhTX-II not reduced; (3) PhTX-II reduced with DTT (1 M).

One myotoxic PLA_2_ was identified and isolated from *P. hyoprora* venom by successive reverse phase high performance liquid chromatography (RP-HPLC) separations. The fractionation of *P. hyoprora* venom using a C18 analytical column produced approximately 20 major peaks (Figure 1). Peak 11 eluting at 62.34% of buffer B and 36.4 min, was positive for PLA_2_ and myotoxic activities and was named PhTX-II PLA_2_. Homogeneity of the purified protein was demonstrated by re-chromatography on an analytical RP-HPLC C18 analytical column, showing the presence of only a peak, (data not shown) and by Tricine SDS-PAGE under non-reducing and reducing conditions revealing a unique band with Mr about 14 kDa each (Figure 1 insert).

**Figure 2:**
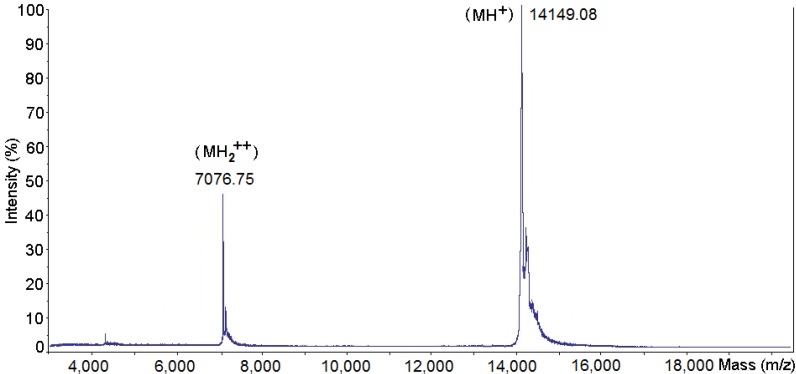
Mass determination of PhTX-II PLA_2_ by mass spectrometry using a Q-Tof Ultima API ESI/MS (TOF MS mode). The MH^+^ and MH_2_^++^ species are shown.

The fraction PhTX-II has a molecular mass of 14149.08 Da, as ascertained by Q-Tof Ultima API ESI/MS (TOF MS mode) mass spectrometry, the MH^+^ and MH_2_^++^ species are also shown (Figure 2). This value of molecular mass was used in calculating the molar concentrations of toxin used in the experiments described below.

### 2.2. Determination of the Amino Acid Sequences of PhTX-II

The alkylated PhTX-II was digested with trypsin and its tryptic peptides were detected and characterized by mass spectrometry. The overall sequences for each peptide were then determined by ESI-MS/MS sequencing. No discrimination between the isobaric residues isoleucine and leucine in any of the sequences reported since they were indistinguishable in low energy CID spectra. Because of the external calibration applied to all spectra, it was also not possible to resolve the 0.036 Da difference between glutamine and lysine residues, except for the lysine that was deduced based on trypsin digestion.

**Table 1:** Sequence obtained by MS/MS based on the alkylated tryptic peptides derived. The peptides were separated and sequenced by mass spectrometry. (Start, End: Amino acid position in the protein sequence. Observed, expected and calculated mass of peptides).

Start	End	Observed	Mr (Expected)	Mr (Calculated)	∆	Sequence
1	7	438.731	875.449	875.486	−0.037	NL/IL/IQFNK(M)
8	15	468.250	934.486	934.515	−0.029	(K)ML/IL/IKETGK(N)
16	33	1079.967	2157.921	2157.918	0.002	(K)NAL/IPFYAFYGCYCGWGGR(G)
34	42	571.690	1140.521	1140.552	−0.030	(R)GKPKDKTDDR(C)
43	53	753.247	1504.479	1504.535	−0.055	(R)CCFVHDCCYGK(L)
54	60	300.403	599.304	599.310	−0.005	(K)L/ITGCPK(W)
61	69	592.800	1183.586	1183.591	−0.004	(K)WDL/IYPYSL/IK(S)
70	77	443.181	884.349	884.406	−0.057	(K)SGYL/ITCGK(G)
78	90	871.806	1741.598	1741.649	−0.051	(K)GTWCEEQL/ICECDR(A)
91	97	404.713	807.411	807.406	0.005	(R)AAAL/ICFR(E)
98	105	498.741	995.468	995.456	0.012	(R)ENL/IDTYNK(Y)
106	115	649.761	1297.508	1297.543	−0.035	(K)YGYMFYPDSR(C)
116	123	483.172	964.330	964.374	−0.043	(R)CKGPSEQC-

Table 1 shows the deduced sequence and measured masses of alkylated peptides obtained. After tryptic digestion of PhTX-II protein, 13 peptides were found. Each sequenced peptide was submitted to the NCBI database, using the protein search program BLAST-p. Using the position matches of the sequenced peptides with phospholipase A_2_ family present in the database, it was possible to deduce their original position on the protein PhTX-II.

**Figure 3:**
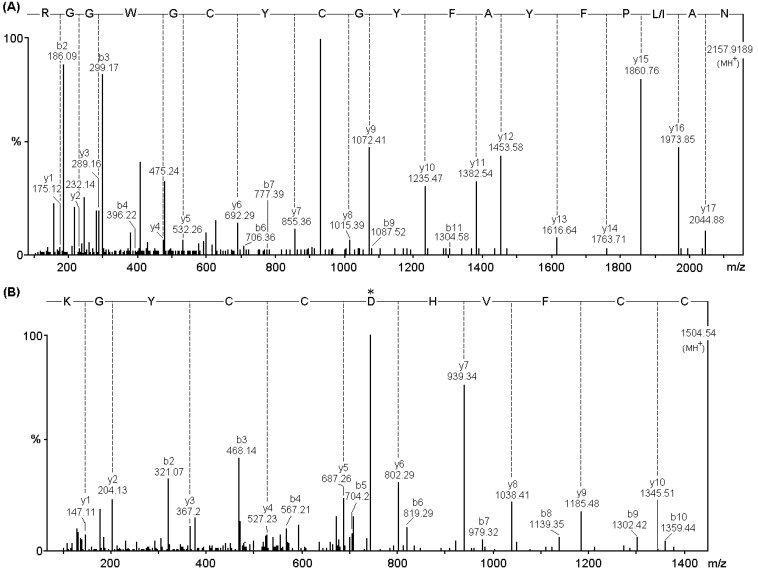
MS/MS spectrum of the tryptic peptides of *m/z* 2157.9189 (**A**) and 1504.54 (**B**); Series of *y* fragment ions of 11-residue-long tryptic peptide (CCFVHDCCYGK) containing the aspartic acid residue at position 49 (*) in the amino acid sequence (**B**) and Ca^2+^-binding loop NAL/IPFYAFYGCYCGWGGR, highly conserved region in the amino acid sequences of PLA_2_ (**A**).

Mass spectra containing the *y*-ion series of the third and fourth peptides and amino acid sequences at positions 16–33 and 43–53, respectively, are shown in Figure 3. The fourth peptide (Figure 3B) has the sequence CCFVHDCCYGK showing an Asp residue at position 49 of the primary structure of the protein, suggesting that this protein belongs to the family of PLA_2_ Asp49 catalytically active. The sequence NAL/IPFYAFYGCYCGWGGR of a third peptide (Figure 3A) show amino acid positions (G)25, (Y)27 (G)29 (G)31 which are part of the calcium-binding, highly conserved domain in PLA_2_ Asp49 and whose presence is required for catalytic activity.

**Figure 4:**
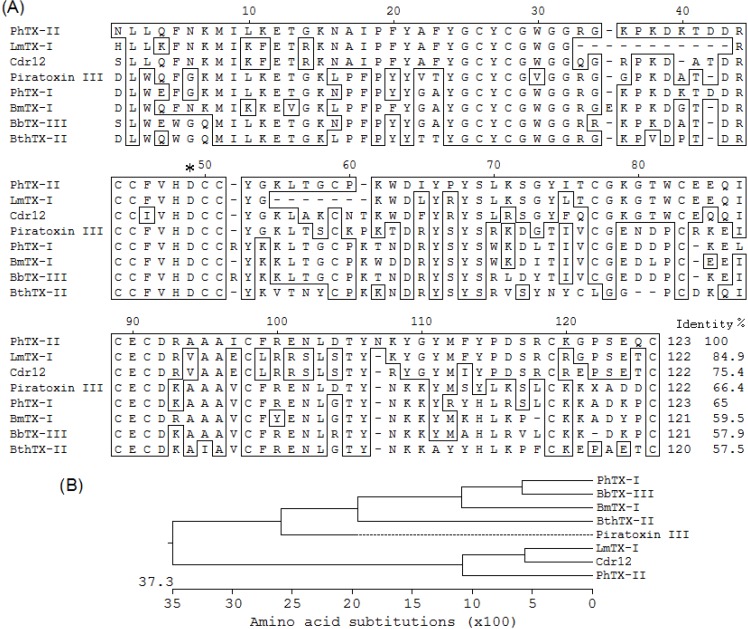
(**A**) Alignment of the amino acid sequence of the PhTX-II PLA_2_ with D49-PLA_2_ by Edit Seq version 5.01© Program (DNASTAR Inc., Madison, WI, USA, 2001). LmTX-I from *Lachesis muta muta* (P0C942.1) [12]; Cdr12 from *Crotalus durissus ruruima* (P0CAS3.1) [13]; Piratoxin III from *Bothrops pirajai* (1GMZ_A) [14]; PhTX-I from *Porthidium hyoprora* [6]; BmTX-I from *Bothrops moojeni* (P0C8M1) [15]; BthTX-II from *Bothrops jararacussu* (2OQD_B) [16]; BbTX-III from *Bothrops brazili* [17]. The asterisk corresponds to Asp49 position; the program introduces gaps to maximize alignments; (**B**) Phylogeny relationship of PhTX-II to other PLA_2_ isoforms statistically evaluated by Bootstrap method.

The amino acid sequence of PhTX-II PLA_2_ was compared with other sequences of myotoxics PLA_2_ already determined and recorded in the database (Figure 4A). By homology PhTX-II is composed of 123 amino acid residues and the arrangement of the cysteines, indicates that PhTX-II belongs to the group IIA PLA_2_. The comparison of PhTX-II amino acid sequence with other published sequences showed a high degree of homology with LmTX-I PLA_2_ (84.9%) and Cdr12 (75.4%). In the case of the PhTX-I PLA_2_ from the same venom, 65% homology was observed. The amino acid sequence analysis revealed that PhTX-II had a strong evolutionary relationship with BbTX-III (Figure 4B).

**Figure 5:**
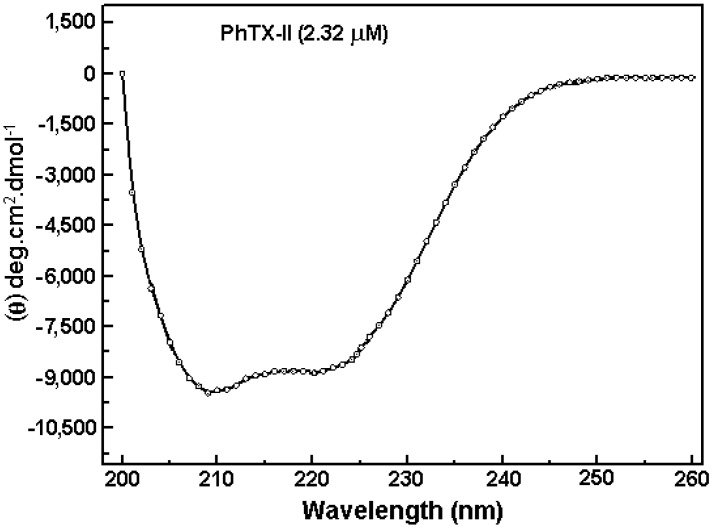
Far-UV circular dichroism spectra of PhTX-II PLA_2_.

Figure 5 shows the CD spectrum of PhTX-II PLA_2_ which demonstrate two negative bands of similar magnitude at 208 and 222 nm (−10.000, −9500 deg.cm^2^.dmol^−1^) and a positive one at ~190 nm, indicating a consistent content of α-helical structures. Based on the CDNN program analysis of the PhTX-II PLA_2_ spectrum, the contents of α-helices, β-sheets and β-turns were 30.5%, 18.2% and 16.9%, respectively.

### 2.3. Activity Measurements of PhTX-II

**Figure 6:**
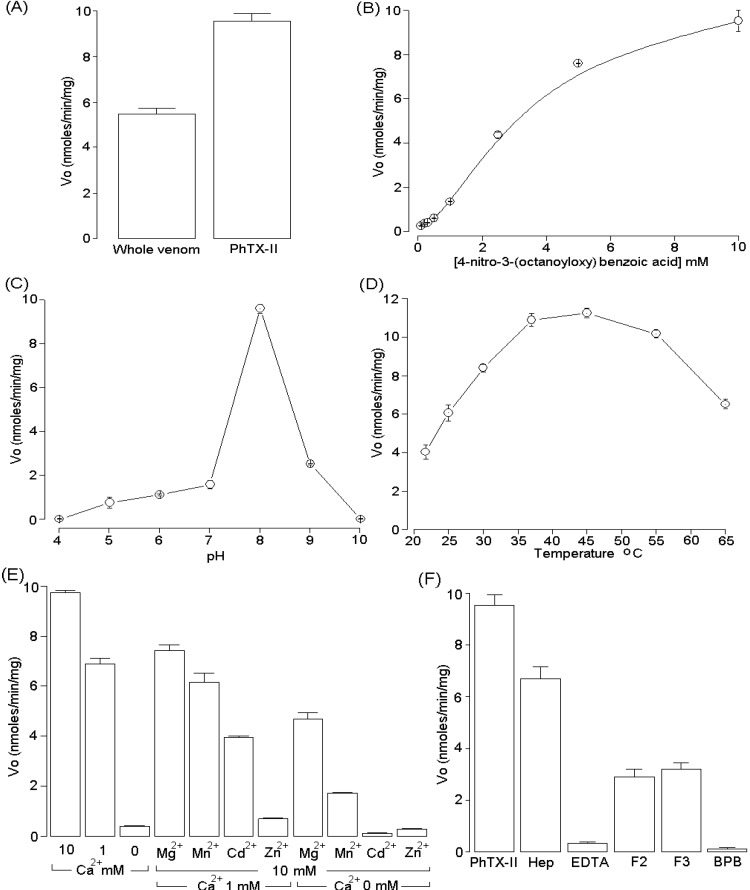
(**A**) PLA_2_ activity of *P. hyoprora* venom and PhTX-II PLA_2_ (**B**) Effect of substrate concentration on the PLA_2_ activity of PhTX-II; (**C**) Effect of pH on the PLA_2_ activity of PhTX-II; (**D**) Effect of temperature on the PLA_2_ activity of PhTX-II; (**E**) Influence of ions (10 mM each) on PLA_2_ activity of PhTX-II in the absence or presence of 1 mM Ca^2+^; (**F**) Effect inhibitory of heparin, EDTA crotapotins (F2 and F3) and chemical modification with BPB on PLA_2_ activity of PhTX-II. In each case, the concentration of PhTX-II was 5 μM. The results of all experiments are the mean ± SEM of three determinations (*p* < 0.05).

The PLA_2_ activity was examined using the synthetic substrate 4-nitro-3-(octanoyloxy) benzoic acid and was higher in PhTX-II PLA_2_ (9.65 ± 0.65 nmols/min/mg) when compared with the whole venom (5.49 ± 0.71 nmols/min/mg) (Figure 6A). Under the conditions used, PhTX-II PLA_2_ showed a discrete sigmoidal behavior (Figure 6B). Maximum enzyme activity occurred at pH 8.0 and the temperature optimum was 40 °C (Figure 6C,D). PhTX-II showed a strict dependence on calcium ions for full activity. The addition of Mg^2+^, Mn^2+^, Cd^2+^ and Zn^2+^ (10 mM) in the presence of low Ca^2+^ concentration (1 mM) decreases the enzyme activity and the substitution of Ca^2+^ by Mn^2+^, Cd^2+^ and Zn^2+^ reduced the activity to levels similar to those in the absence of Ca^2+^ (Figure 6E). The incubation of PhTX-II with crotapotins F2 and F3 from *C. d. collilinetaus* and EDTA diminished the enzymatic activity; heparin did not significantly inhibit this activity. On the other hand, the enzymatic activity of PhTX-II PLA_2_ was almost completely abolished by *p*-bromophenacyl bromide (BPB) (Figure 6F).

### 2.4. Pharmacological Activities of PhTX-II

**Figure 7:**
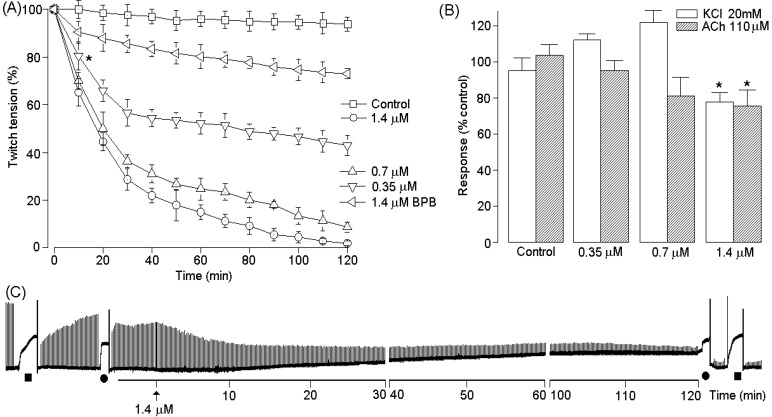
Neuromuscular blockade of chick *biventer cervicis* preparations incubated with PhTX-II PLA_2_ (0.35, 0.7, 1.4 μM and modified with BPB) at 37 °C (**A**). Panel B shows the effect of PhTX-II PLA_2_ on muscle contractures evoked by KCl and acetylcholine (ACh). Panels C shows a representative recording from a preparation treated with 0.7 μM of PhTX-II PLA_2_. Tissue responses to KCl (■ 20 mM) and acetylcholine (ACh, ● 110 μM) were obtained before and after toxin addition. The points are the mean ± SEM of six experiments. * (*p* < 0.05) compared to the twitch-tension before toxin addition.

**Figure 8:**
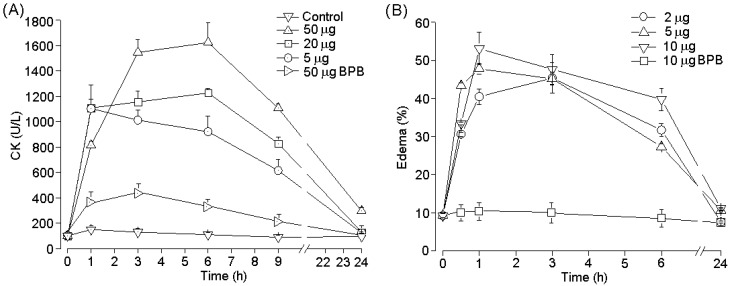
Time-course of the increments in plasma CK activity after intramuscular injection of PhTX-II PLA_2_ (5.20, 50 μg and modified with BPB/100 μL) in mice. Controls were injected with 100 μL of PBS. At different times, blood was collected, and serum levels were measured. Values are means ± SEM of five mice at each time point (**A**); Edema-forming activity of PhTX-II PLA_2_ (2.5, 10 μg and modified with BPB/50 µL) in mice. Edema by toxin was injected s.c. in the footpad of mice. At various time intervals the increase in footpad volume, as compared to controls, was expressed as percent edema (**B**). Each point represents the mean ± SEM of five animals.

PhTX-II PLA_2_ produced irreversible time- and concentration-dependent neuromuscular blockade in indirectly stimulated *biventer cevicis* preparations, with complete blockade occurring after 110 ± 2 min at the highest concentration (1.4 μM); The times required for 50% blockade were 53.47 ± 3.2, 26.7 ± 2.4 and 17.9 ± 6.5 min for PhTX-II concentrations of 0.35, 0.7 and 1.4 μM, respectively (Figure 7A). Pretreatment of PhTX-II with BPB decreased dramatically the neuromuscular blockade by this toxin (Figure 7A). At concentrations of 0.35 and 0.7 μM, there were no consistently signiﬁcant changes in the contractures to exogenous ACh and KCl after complete neuromuscular blockade by PhTX-II; however, incubation with 1.4 μM decreased the muscle contractures to exogenous ACh and KCl, with 75.3% ± 9.4% and 77.7% ± 5.30% of the response remaining at the end of the experiment, respectively (Figure 7B). Figure 7C shows a representative recording of the neuromuscular blockade produced by PhTX-II PLA_2_ (1.4 μM) under indirect stimulation at 37 °C. The injection of PhTX-II PLA_2_ by the intramuscular (i.m.) route induce significant increment of plasma CK levels at doses 5, 20 and 50 µg. Time-course analysis showed a maximum increase in plasma CK 3–6 hours after injection, returning to normal by 24 h (Figure 8A). Three hours after treatment started, the myotoxic effect of PhTX-II PLA_2_ was reduced in 82%, by treatment with BPB (Figure 8A). On the other hand, PhTX-II PLA_2_ (2, 5, and 10 μg/paw) also induced moderate footpad edema, compared to PBS-injected animals. Edema reached its highest point after 1 h, and receded to normal levels after 24 h, but this effect was not observed in case of BPB-inactivated enzyme (Figure 8B).

## 3. Discussion

The presence of abundant PLA_2_ from snake venom as multiple isoenzymes is a common observation [9,18]. These isoenzymes in addition to their involvement in the digestion of prey, exhibit a wide variety of pharmacological/toxic effects by interfering in normal physiological processes of prey/victims [19]. We have previously described the biochemical characterization and some biological activities of PhTX-I, a basic PLA_2_ isolated from *P. hyoprora* that induces muscle damage in mice (leading to CK release) and is pro-inﬂammatory, causing edema [6]. However, many more components of this venom need to be isolated, and to be characterized [10]. This work describes the biochemical/pharmacological characterization of PhTX-II, a PLA_2_ isolated from *P. hyoprora* venom by a single chromatographic step, including reverse-phase chromatography (Figure 1).

The PhTX-II PLA_2_ is an enzyme composed of a unique polypeptidic chain revealed by Tricine SDS-PAGE with molecular mass of 14,149.08 Da confirmed by mass spectrometry (Figure 2). This value is similar to other isolated myotoxic PLA_2_ from snake venom [20,21,22].

PhTX-II is composed of 123 amino acid residues and the arrangement of the cysteines indicates that PhTX-II belongs to group IIA PLA_2_. As Leu (L) and Ile (I) are indistinguishable with MS/MS analysis, it is essential to determine completely the primary structure of PhTX-II PLA_2_ by protein sequencer or cDNA analysis. Amino acid sequence of PhTX-II showed the presence of Asp at position 49 (Figure 4), this finding place for the PhTX-II within the catalytically active enzymes, consistent with a lot of PLA_2_ purified from viperid snake venoms where Asp49 appears so far to be an absolutely conserved position [12,23,24,25]. The presence of an aspartic residue at 49 position allow the interaction with the ion calcium (Ca^2+^-dependence) and together with Tyr28, Gly30 and Gly31 form the Ca^2+^-binding loop [26]. The His48 is in a key position since it participates in the PLA_2_ active site as a catalytic residue together with Tyr52 and Asp99 [27]. PhTX-II PLA_2_ displays residues forming the catalytic network and the Ca^2+^-binding loop (Figure 3A,B), reflected in the high catalytic activity displayed by the enzyme.

In addition, PLA_2_ enzymes present a hydrophobic channel that provides access to the catalytic site ((L)2, (W/F)3, (I)9, (Y)22, (C)29, (C)45 and (A)93) are important for the enzymatic activity and biological profile of snake venom PLA_2_ [28]. Tyr73 and Lys56 also indirectly participate in the stabilization of the catalytic site of Asp49 PLA_2_ [29]. All these amino acids are conserved in the PhTX-II PLA_2_, allowing the lipidic substrate access to the catalytic site of the enzyme.

The comparison of PhTX-II amino acid sequence with other published sequences (Figure 4A) showed a high degree of homology with LmTX-I PLA_2_, Cdr12, and Piratoxin III. Curiously, PhTX-I-another PLA_2_ isolated from *P. hyoprora* venom [6] share 65% homology with PhTX-II, revealing evolutionary distance greater than the other PLA_2_ isoforms (Figure 4B). These observations probably are due to that exons in PLA_2_ genes mutate faster than introns, and this accelerated evolution of exons plays a significant role in the emergence of new isoenzymes by altering the target specificity. Similar results were found with *Bothrops leucurus* and *Bothrops asper* [9,20,24].

After determining the primary structure, PhTX-II was analyzed by CD to check the status folding through the quantification of secondary structure. Examination of PhTX-II PLA_2_ by CD spectroscopy indicated a high content of alpha-helical structures, similar to the known structure of secreted phospholipase IIA group [30], suggesting a similar folding. The contribution of 30.5% of the α-helix structure (Figure 5) is consistent with the crystal structure of PLA_2_ snake venom, where the presence of three α-helices is demonstrated, which are part of the N-terminus and the hydrophobic channel of access to catalytic site, these regions being related with important roles for catalysis as well as pharmacological activities [29].

The enzymatic activity of PhTX-II PLA_2_ was confirmed using the 4-nitro-3-octanoyloxy-benzoic acid as substrate, and showed a high catalytic activity (9.65 ± 0.65 nmoles/min/mg) (Figure 6A) when compared with Bj-V (3.49 ± 0.227 nmoles/min/mg) an Asp49 basic and myotoxic PLA_2_ purified from *B. jararacusuu* venom [31]. The optimum temperature was around 37 °C, but at 40–45 °C, the PhTX-II PLA_2_ activity did not present a huge decrease (Figure 6D). Like other PLA_2_, PhTX-II is relatively resistant to heat [12,19]. Highest enzymatic activity occurs at pH 8 (Figure 6C). At low concentrations of substrate, PhTX-II showed a discrete sigmoidal behavior, as well as BbTX-III and Bbil-TX (purified from *Bothrops brazili* and *Bothriopsis bilineata*, respectively) that showed similar behavior towards this non-micellar substrate [17,21]. We can suggest that the specificity of the enzyme/substrate interaction for PhTX-II pointed to a structural resemblance of this enzyme with those PLA_2_ that also had similar behavior with the substrate mentioned above.

The dependence on Ca^2+^ ions for full enzymatic activity of PLA_2_ was previously described by Scott *et al.* [26]. PhTX-II was a typical Ca^2+^-dependent PLA_2_ that was less active in the presence of other divalent cations (Mg^2+^, Mn^2+^, Cd^2+^, Zn^2+^) (Figure 6E). This can be explained by coordination geometries assumed by the tetrahedral intermediate due to the presence of Ca^2+^ ions, which determine the electrophilic behavior of the catalytic site [29]. However, Mg^2+^ ions support a significant catalytic activity. These results can be attributed to differences in coordination geometry and indicate a noteworthy plasticity of the active site environment.

Incubation of PhTX-II with EDTA diminished the enzymatic activity (Figure 6F), since PLA_2_ enzymes require Ca^2+^ for their activity, chelators such as EDTA inhibit their enzymatic activity and which affects normal functions of the PLA_2_ enzymatic system [32]. The crotapotins are pharmacologically inactive and non-catalytic acidic proteins that specifically bind to the PLA_2_ of different sources (pancreas, bee, snake venom) and inhibit its catalytic activity [33]. F2 and F3 crotapotins from *C. d. collilineatus* significantly inhibited the enzymatic activity of PhTX-II by approximately 60% (Figure 6F). These results are in agreement with reports of BjIV PLA_2_ from *B. jararacussu*, which was inhibited by 50% in its catalytic activity by F7 crotapotins from *C. d. terrificus*, F3 and F4 from *C. d. collilineatus* and *C. d. cascavella* [31]. Heparin slightly decreased enzymatic activity of PhTX-II; similarly, this polyanionic compound also became a negative allosteric modulator of PLA_2_ of *C. d. cascavella* [34].

The treatment of PhTX-II PLA_2_ by BPB resulted in complete loss of their catalytic activity (Figure 6F). The His48 is in a key position since it participates in the PLA_2_ active site as a catalytic residue [29], since the enzymatic activity of the PhTX-II was completely abolished after this modification, His48 was likely the residue modified, because this amino acid is part of the catalytic triad of this protein family. His-modified enzymes are suitable for both *in vitro* and *in vivo* systems in order to study the role of the enzymatic activity on the PLA_2_ pharmacological profiles [11,35,36].

Skeletal muscle necrosis is a frequent and serious consequence of snakebite envenoming, potentially leading to permanent loss of tissue and disability [37]. In a previous work, we showed that *P. hyoprora* venom cause intense local myotoxicity, inflammation and cytotoxicity [6]. Neuromuscular paralyzing, edematogenic and myotoxic activities were investigated, in order to understand the contribution of PhTX-II PLA_2_ in the myotoxic effect of *P. yoprora*.

As shown here, PhTX-II PLA_2_ causes time- and concentration-dependent neuromuscular blockade in avian nerve–muscle preparations. With 1.4 and 0.7 μM of PhTX-II, fifty percent blockade occurred before 30 min, while blockade with minor concentration (0.35 μM) occurred after 50 min (Figure 7A). In addition to the neuromuscular blockade, slight muscle contracture (increase in baseline tension) was observed with the highest concentration (1.4 μM) (Figure 7C). Moreover, neuromuscular block produced by PhTX-II at the same concentrations was accompanied by diminution of responses to potassium (KCl) and acetylcholine (ACh) (Figure 7B). These observations suggest damage caused to muscle membrane and nicotinic receptors in the post-synaptic membrane. The presence of myotoxic components in the venom are shown to reduce the response of a skeletal muscle to direct electrical stimulation or exposure to high concentrations of K^+^ and/or initiate muscle contracture [38].

Incubation with BPB inhibited enzymatic activity of PhTX-II and dmarkedly decreased toxin-induced neuromuscular blockade (around 89%) in chick *biventer cervicis* preparations (Figure 7A) strengthening the hypothesis that phospholipid enzymatic hydrolysis is involved in this effect. Similarly, neurotoxic activity was inhibited almost completely after alkylation of His48 of Bbil-TX PLA_2_ from *Bothriopsis bilineata*, Cdc-9 and Cdc-10 PLA_2_ from (*Crotalus durissus cumanensis* and PhTX-I from *P. hyoprora* [11,39,40].

It was pointed out by Francis *et al.*, [41] that myotoxic proteins from snake venoms have a set of three or four tyrosine residues between positions 114 and 124; PhTX-II PLA_2_ have four tyrosine residues in this region, which is reflected in their high myotoxicity. PhTX-II markedly increased plasmatic creatin kinase (CK) levels after being injected into the gastrocnemius muscle of mice; this increase in CK shows severe muscle damage in muscle fibers injected with the toxin, leading to alocal myotoxic effect (Figure 8A). Theplasmatic increased of CK observed at different time intervals when there is phospholipid hydrolysis suggests that phospholipid degradation is an initial event that leads to membrane perturbation, resulting initially in increased permeability to ions and small molecules, such as Ca^2+^, K^+^ and ATP, and afterwards to large membrane lesions through which proteins such as CK are released [42].

Previous studies suggest that catalytic activity plays a key role in the myotoxicity induced by Asp49 PLA_2_ myotoxins [36]. However, after chemical modification with BPB, PhTX-II PLA_2_ yielded 18% myotoxic activity in mice, thus PhTX-II-induced local myotoxicity is dependent not only on enzymatic activity.

The Asp49 PLA_2_ myotoxins, besides causing muscle necrosis, have been shown to promote marked local inflammatory events in several experimental models [21,43,44]. The sub plantar injection of this PhTX-II PLA_2_ also caused mouse paw edema (Figure 8B). In addition, the treatment of PhTX-II by BPB resulted in complete loss of their catalytic activity that was accompanied by loss of their edematogenic effect. The mechanism by which catalytically active PLA_2_ induce edema may be explained by phospholipid hydrolysis, probably due to liberation of precursors of a variety of eicosanoids and platelet activator factors [33]. Thus, MT-III a Asp49 PLA_2_ from *Bothrops asper* induces cyclooxygenase-2 expression and prostaglandin E_2_ production via activation of NF-κB, p38MAPK, and PKC in macrophages [44].

## 4. Materials and Methods

### 4.1. Venom and Reagents

The venom and the solvents (HPLC grade), 4-nitro-3-(octanoyloxy) benzoic acid, sequence grade bovine pancreatic trypsin, 2,4’-Dibromoacetophenone (BPB) and other reagents were from Sigma Chemical Co. (St. Louis, MO, USA). The animals and research protocols used in this study followed the guidelines of the Ethical Committee for use of animals of ECAE-IB-UNICAMP SP, Brazil (protocol number 1860-1) and international law and policies.

### 4.2. Purification of PhTX-II

The PhTX-II PLA_2_ from *P. hyoprora* venom was purified by reverse phase HPLC using 500 mg of whole venom, according to the method described by Ponce-Soto *et al.*, [45]. Briefly, 5 mg of whole venom was dissolved in 200 µL of buffer A (0.1% TFA) and centrifuged at 4500 *g*, the supernatant was then applied to a µ-Bondapack C18 column (0.78 × 30 cm; Waters 991-PDA system), previously equilibrated with buffer A for 15 min. The elution of the protein was then conducted using a linear gradient (0%–100%, *v*/*v*) of buffer B (66.5% Acetronitrile in buffer A) at a constant flow rate of 1.0 mL/min. The chromatographic run was monitored at 280 nm of absorbance and the PLA_2_ active fractions (PhTX-II) were collected, lyophilized and used for biochemical/pharmacological characterization. To verify the degree of homogeneity, re-chromatography of PhTX-II was performed by the same chromatography system used in the fractionation step (reverse phase HPLC). The protein was concentrated by ultrafiltration through a YM-3 Amicom membrane and washed with water or ammonium bicarbonate 0.05 M, pH 8.0 followed by lyophilization.

### 4.3. Electrophoresis

Tricine SDS-PAGE in a discontinuous gel and buffer system was used to estimate the molecular mass of the PhTX-II PLA_2_, under reducing and non-reducing conditions [46]. The molecular mass markers used were (in kDa): phosphorylase B—94, albumin—67, ovalbumin—43, carbonic anhydrase—30, soybean trypsin inhibitor—20 and lysozyme—14.

### 4.4. Determination of the Molecular Mass of the Purified Protein by Mass Spectrometry

An aliquot (4.5 μL) of the PhTX-II PLA_2_ was inject by C18 (100 μm × 100 mm) RP-UPLC (nanoAcquity UPLC, Waters Corporation, Milford, MA, USA) coupled with nanoelectrospray tandem mass spectrometry on a Q-Tof Ultima API mass spectrometer (MicroMass/Waters Corporation, Milford, MA, USA) at a flow rate of 600 mL/min. The gradient was 0%–50% acetonitrile in 0.1% formic acid over 45 min. The instrument was operated in MS continuum mode and the data acquisition was from *m*/*z* 100–3000 at a scan rate of 1 s and an interscan delay of 0.1 s. The spectra were accumulated over about 300 scans and the multiple charged data produced by the mass spectrometer on the *m*/*z* scale were converted to the mass (molecular weight) scale using maximum-entropy-based software (1) supplied with Masslynx 4.1 software package. The processing parameters were: output mass range 6,000–20,000 Da at a “resolution” of 0.1 Da/channel; the simulated isotope pattern model was used with the spectrum blur width parameter set to 0.2 Da and the minimum intensity ratios between successive peaks were 20% (left and right). The deconvoluted spectrum was then smoothed (2 × 3 channels, Savitzky Golay smooth) and the mass centroid values obtained using 80% of the peak top and a minimum peak width at half height of 4 channels.

### 4.5. Analysis of Tryptic Digests

The PhTX-II PLA_2_ was reduced (DTT 5 mM for 25 min to 56 °C) and alkylated (Iodoacetamide 14 mM for 30 min) prior to the addition of trypsin (Promega’s sequencing grade modified). After trypsin addition (20 ng/μL in Ambic 0.05 M), the sample was incubated for 16 h at 37 °C. To stop the reaction, formic acid 0.4% was added and the sample centrifuged at 2500 rpm for 10 min. The pellet was discarded and the supernatant dried in a speed vac. The resulting peptides were separated by C18 (100 μm × 100 mm) RP-UPLC (nanoAcquity UPLC, Waters Corporation, Milford, MA, USA) coupled with nanoelectrospray tandem mass spectrometry on a QTof Ultima API mass spectrometer (Micro Mass/Waters Corporation, Milford, MA, USA) at a flow rate of 600 nL/min. Before performing a tandem mass spectrum, an ESI/MS mass spectrum (TOF MS mode) was acquired for each HPLC fraction over the mass range of 100–2000 *m*/*z*, in order to select the ion of interest; subsequently, these ions were fragmented in the collision cell (TOF MS/MS mode).

Raw data files from LC-MS/MS runs were processed using Masslynx 4.1 software package (Waters) and analyzed using the MASCOT search engine version 2.3 (Matrix Science Ltd., Mascot Server version 2.3, London, UK, 2012) against the snakes database, using the following parameters: peptide mass tolerance of ±0.1 Da, fragment mass tolerance of ±0.1 Da, and oxidation as variable modifications in methionine and trypsin as enzyme.

### 4.6. Circular Dichroism

Circular dichroism (CD) spectra of PhTX-II PLA_2_ was recorded with a JASCO model J-720-ORD 306 spectropolarimeter equipped with a thermoelectric sample temperature controller (Peltier system) following standard procedures previously described [47]. After centrifugation at 4000 g for 5 min, samples (1–4 μM protein in 10 mM sodium phosphate, pH 8) were transferred to a 10-mm path-length quartz cuvette. Circular dichroism spectra in the wavelength range 260–200 nm were collected, using a bandwidth of 1 nm and a response time of 1 s. Data collection was performed at 25 °C with 50 nm/min scanning speed. At least 10 scans were accumulated for each sample, and all spectra were corrected by subtraction of buffer blanks. The estimation of secondary structure elements was performed using the CDNN Deconvolution software,version 2.1 (Applied Photophysics, Leatherhead, Surrey, UK, 2011) and Origin^®^ 7.5 (version 7.5, OriginLab Corporation, Wellesley Hills, MA, USA, 2003) was used for graphics and analysis.

### 4.7. Chemical Modifications

Modification of His residues with *p*-Bromophenacyl Bromide (BPB) was carried out as previously described [35]. Briefly, 3 mg of PhTX-II PLA_2_ were dissolved in 1 mL of 0.1 M Tris-HCl containing 0.7 mM EDTA (pH 8.0) and 150 µL of BPB (1.5 mg/mL, in ethanol), and the mixture incubated for 24 h at 25 °C.

### 4.8. PLA_2_ Activity

PLA_2_ activity was measured using the assay described by Cho and Kezdy, [48] and Holzer and Mackessy, [49] modified for 96-well plates. The standard assay mixture contained 200 µL of buffer (10 mM Tris-HCl, 10 mM CaCl_2_ and 100 mM NaCl, pH 8.0), 20 µL of substrate 4-nitro-3-(octanoyloxy) benzoic acid (3 mM), 20 µL of water and 20 µL of PhTX-II PLA_2_ or modified with BPB (1 mg/mL) in a final volume of 260 µL. After adding proteins (20 µg) the mixture was incubated for up to 40 min at 37 °C, measuring absorbance at intervals of 10 min. The enzyme activity, expressed as the initial velocity of the reaction (*V_o_*), was calculated based on the increase of absorbance after 20 min. The pH and optima temperature of the PhTX-II PLA_2_ were determined by incubating the enzyme in four buffers of different pH values (4–10) and in Tris-HCl buffer, pH 8.0, at different temperatures, respectively. The effect of substrate concentration (40, 20, 10, 5, 2.5, 1.0, 0.5, 0.3, 0.2 and 0.1 mM) on enzyme activity was determined by measuring the increase of absorbance after 20 min of incubation in Tris-HCl buffer, pH 8.0 at 37 °C. All assays were done in triplicate and the absorbances at 425 nm were measured with a VersaMax 190 multiwell plate reader (Molecular Devices, Sunnyvale, CA, USA). The protein concentration of PhTX-II was determined by the Bradford protein assay.

### 4.9. Inhibition

The inhibitory effect of EDTA or low molecular weight heparin from porcine intestinal (Mr 6000 Da) on pharmacological and enzymatic activities of PhTX-II PLA_2_ were assessed by incubating the enzyme with 1 mM solution of this chelating agent or a heparin : toxin molar ratio of 2:1 for 30 min at 37 °C. The inhibition of PLA_2_ activity of PhTX-II by crotapotins F2 and F3 from *Crotalus durissus collilinetaus* also was evaluated by incubating the two proteins (1:1, *w*/*w*) for 30 min at 37 °C and then assaying the residual enzyme activity.

### 4.10. Chick Biventer Cervicis Muscle Preparation (BCP)

Animals were anesthetized with halothane and sacrificed by exsanguination. The *biventer cervicis* muscles were removed and mounted under a tension of 0.5 g, in a 5 mL organ bath (Automatic organ multiple-bath LE01 Letica Scientific Instruments, Barcelona, Spain) at 37 °C containing aerated (95% O_2_-5% CO_2_) Krebs solution (pH 7.5) of the following composition (mM): NaCl 118.70, KCl 4.70, CaCl_2_ 1.88, KH_2_PO_4_ 1.17, MgSO_4_ 1.17, NaHCO_3_ 25.00 and glucose 11.65. Contracture to exogenously applied acetylcholine (ACh; 110 μM for 60 s) and KCl (20 mM for 130 s) was obtained in the absence of field stimulation, before and after the addition of a single dose of PhTX-II PLA_2_ (0.35, 0.7 and 1.4 μM). A bipolar platinum ring electrode was placed around the tendon, which runs around the nerve trunk supplying the muscle. Indirect stimulation was performed with a (MAIN BOX LE 12404 Panlab s.l. Powerlab AD Instruments Barcelona, Spain) stimulator (0.1 Hz, 0.2 ms, 3–4 V). Muscle contractions and contractures were isometrically recorded by force-displacement transducers (Model MLT0201 Force transducer 5 mg–25 g Panlab s.l. AD Instruments Pty Ltd., Barcelona, Spain) connected to a PowerLab/4SP (OUAD Bridge AD Instruments, Barcelona, Spain) [50].

### 4.11. Myotoxic Activity

Groups of five Swiss mice (18–20 g) received an intramuscular injection (i.m.) of variable amounts of PhTX-II PLA2 (5, 20 and 50 μg) dissolved in 100 µL of PBS, in the gastrocnemius. A control group received 100 µL of PBS (0.12 M NaCl, 0.04 M sodium phosphate, pH 7.2). At different intervals of time (1, 3, 6, 9 and 24 h) blood was collected from the tail into heparinized capillary tubes, and the plasma creatine kinase (CK; EC 2.7.3.2) activity was determined by a kinetic assay (Sigma 47-UV, Sigma-Aldrich, St. Louis, MO, USA). Activity was expressed in *U*/*L* one unit defined as the phosphorylation of 1 µmol of creatine/min at 25 °C [51].

### 4.12. Edema-Forming Activity

The ability of PhTX-II PLA_2_ to induce edema was studied in groups of five Swiss mice (18–20 g). Fifty µL of phosphate-buffered saline (PBS; 0.12 M NaCl, 0.04 M sodium phosphate, pH 7.2) with toxins (2, 5 and 10 µg/paw) were injected in the sub plantar region of the right footpad. The left footpad received 50 µL of PBS, as a control. The paw volume was evaluated plethysmographically (Model 7140 Plethysmometer Ugo Basile, Gemonio, Varese, Italy), immediately before the injection (basal) and at selected intervals of time (0.5, 1, 3, 6, 9 and 24 h). Edema-forming activity was expressed as the percentage of the increase in volume of the right foot pad in comparison to the left foot pad (control). The equation for calculation of the percentage of edema in toxins injected paw was: % edema = (((Tx × 100)/ To) − 100). Where Tx is the edema (volume) measured at each time interval and To is the volume of the paw (intact, zero time before toxins injection). The percentage of edema calculated was subtracted from the matched values at each time point in the saline injected hind paw (control) [52].

### 4.13. Statistical Analyses

Results were reported as mean ± SEM. The significance of differences among means was assessed by analysis of variance followed by Dunnett’s test, when several experimental groups were compared with the control group. Differences were considered statistically significant if *p* < 0.05.

## 5. Conclusions

In conclusion, we have identified and characterized the PhTX-II enzyme, a new Asp-49 PLA_2_ monomeric isolated from *P. hyoprora* venom. PhTX-II PLA_2_ causes neuromuscular blockade in avian neuromuscular preparations with a significant direct action on skeletal muscle function and additionally, also induced local edema and myotoxicity in mice. The enzymatic activity of PhTX-II contributes to this neuromuscular blockade and the induced local myotoxicity is dependent not only on enzymatic activity. Taken together, these results confirm the importance of the contribution of PhTX-II PLA_2_ in the envenomation by this snakebite, where this isoform is able to trigger higher myotoxicity and evidence structural differences which are of particular value to understanding the complex structure-function relationships that govern this highly diverse group of snake venom proteins.
